# Adenosine A_2A_ Receptors Mediate Resveratrol-Induced Nrf2 Activation and Cytoprotection Against Oxidative Stress in HeLa Cells

**DOI:** 10.3390/ph19060853

**Published:** 2026-05-29

**Authors:** Clara Fructuoso González, Alejandro Sánchez-Melgar, José Luis Albasanz Herrero, Mairena Martín López

**Affiliations:** 1 Department of Inorganic and Organic Chemistry and Biochemistry, Faculty of Chemical Sciences and Technologies, University of Castilla-La Mancha, Avenida Camilo José Cela 10, 13071 Ciudad Real, Spain; clara.fructuoso@alu.uclm.es (C.F.G.); alejandro.sanchez@uclm.es (A.S.-M.); mairena.martin@uclm.es (M.M.L.); 2 Faculty of Nursing of Ciudad Real, University of Castilla-La Mancha, Avenida Camilo José Cela 14, 13071 Ciudad Real, Spain; 3 Institute of Biomedicine (IB-UCLM), University of Castilla-La Mancha, Almansa s/n, 02071 Albacete, Spain; 4 Instituto de Investigación Sanitaria de Castilla-La Mancha (IDISCAM), Finca de la Peraleda s/n, 45071 Toledo, Spain; 5 Faculty of Medicine of Ciudad Real, University of Castilla-La Mancha, Camino Moledores s/n, 13071 Ciudad Real, Spain

**Keywords:** resveratrol, adenosine A_2A_ receptors, Nrf2, oxidative stress

## Abstract

**Background/Objectives:** Oxidative stress is a major contributor to cellular injury in many pathological conditions, including neurodegenerative disorders. Resveratrol, a natural polyphenol with antioxidant properties, has been proposed as a cytoprotective compound, although the molecular mechanisms underlying its effects remain incompletely understood. Here, we investigated whether the protective action of resveratrol against hydrogen peroxide-induced oxidative stress is mediated by adenosine receptor signalling and activation of the Nrf2 pathway in HeLa cells. **Methods:** Cells were treated with resveratrol alone or in combination with selective adenosine receptor antagonists and oxidant challenge, and cell viability, ROS production, receptor involvement, and Nrf2 expression and localization were analyzed. **Results:** Resveratrol at a non-toxic concentration significantly protected HeLa cells against oxidative damage, reduced ROS accumulation, promoted Nrf2 nuclear translocation and gene expression, and enhanced the gene expression of antioxidant enzymes such as SOD1, catalase, HO-1, and NQO1. Pharmacological blockade of the A_2A_ receptor prevented this protective effect, whereas the inhibition of A_1_ and A_3_ receptors enhanced it and avoided the increased SOD1, catalase, HO-1, and NQO1 gene expression promoted by resveratrol alone. Moreover, A_2A_ antagonism was associated with reduced PKA levels, consistent with the involvement of the cAMP/PKA signalling axis. **Conclusions:** Taken together, these observations support a model in which adenosine A_2A_ receptor signalling contributes to resveratrol-associated cytoprotection and Nrf2 activation in a human non-neuronal cell model. Our findings therefore provide mechanistic insight into resveratrol–adenosine receptor interactions and generate hypotheses to be tested in disease-relevant neuronal systems.

## 1. Introduction

Oxidative stress arises from an imbalance between the production of reactive oxygen species (ROS) and reactive nitrogen species (RNS) and cellular antioxidant defences, contributing to a wide spectrum of pathologies, including neurodegenerative diseases, cardiovascular disorders (atherosclerosis, hypertension), diabetes mellitus, cancer progression, and chronic inflammatory conditions such as arthritis [1,2,3,4].

ROS, including hydrogen peroxide (H_2_O_2_), superoxide anions, and hydroxyl radicals, are byproducts of mitochondrial respiration and enzymatic reactions [5]. While physiological ROS levels serve signalling functions [6], the excessive accumulation of damages in lipids, proteins, and DNA, exacerbating cytotoxicity in different pathologies, including those such as Huntington’s disease, Alzheimer’s disease (AD), and Parkinson’s disease [7,8].

Brain tissue is particularly vulnerable to oxidative damage, likely due to its high oxygen consumption [9], which leads to ROS-elicited destructive consequences in DNA and proteins involved in many brain processes [8]. However, oxidative stress is also a key contributor to redox imbalance and cellular injury in non-neuronal systems, including cancer-derived cell lines such as HeLa cells [10]. For this reason, HeLa cells can serve as a tractable human model to dissect conserved cytoprotective pathways such as Nrf2 signalling and adenosine receptor modulation [11], while recognizing that direct relevance to neurodegenerative diseases requires validation in neuronal or glial systems [6,9].

The nuclear factor erythroid 2-related factor 2 (Nrf2) is a transcriptional factor that orchestrates the primary cellular response to oxidative stress by inducing the expression of antioxidant and detoxifying enzymes (e.g., HO-1, NQO1, SOD, and GST) through antioxidant response elements (AREs) [12]. Under homeostatic conditions, Nrf2 is sequestered by Kelch-like ECH-associated protein 1 (Keap1) in the cytosol, targeting it for proteasomal degradation. Oxidative stress disrupts Keap1-Nrf2 binding, enabling Nrf2 nuclear translocation and transcriptional activation [13]. Nrf2 pathway dysfunction underlies age-related oxidative vulnerability and is implicated in neurodegenerative progression [14,15].

Resveratrol (RSV) is a polyphenolic stilbene (3,5,4′-trihydroxy-trans-stilbene) found in grapes, berries, and red wine [16]. RSV has been reported to activate Nrf2 through multiple convergent mechanisms in other models. At low micromolar concentrations, RSV can promote Nrf2 stabilization by disrupting Keap1 interactions, enhance Nrf2 phosphorylation via PI3K/Akt and AMPK/p38 MAPK pathways, and stimulate SIRT1-dependent deacetylation for optimal transcriptional activity [17,18,19]. In the present work, we did not directly assess these pathways, but we focus on the involvement of adenosine receptors and Nrf2 in HeLa cells. These actions upregulate endogenous antioxidant defences while RSV itself exhibits a mild ROS-scavenging capacity at higher doses (>50 μM) [20]. Preclinical studies support RSV’s cytoprotective potential across cellular and animal models of oxidative injury, positioning it as a candidate for oxidative stress-related disorders [21,22]. However, clinical translation remains challenging due to pharmacokinetic limitations, biphasic dose–response relationships, and the age-dependent attenuation of Nrf2 signalling [23,24,25,26].

Adenosine receptors (AdoRs), a family of G protein-coupled receptors (A_1_, A_2A_, A_2B_, and A_3_), modulate diverse physiological processes including inflammation, neurotransmission, and cellular metabolism [27]. RSV has been reported to interact with all AdoRs subtypes with micromolar affinity, behaving as a non-selective modulator rather than a strictly selective agonist [28]. A_2A_ and A_2B_ (Gs-coupled) receptors stimulate adenylyl cyclase (AC) and cAMP/PKA signalling, promoting glutamate release and pro-inflammatory cytokine production in microglia and astrocytes. Conversely, A_1_ and A_3_ (Gi-coupled) receptors inhibit AC, reducing excitotoxicity and inflammation [29,30]. A_2A_ receptor overexpression occurs in AD brain regions and correlates with amyloid-β accumulation and cognitive decline [31,32]. Selective A_2A_ antagonists (e.g., istradefylline, SCH58261) ameliorate AD pathologies in preclinical models by reducing neuroinflammation, tau hyperphosphorylation, and synaptic dysfunction [33,34,35].

In tumours, AdoRs also play complex roles in modulating cell proliferation, immune evasion, and tumour microenvironment dynamics, with A_2A_ and A_2B_ receptors often promoting immunosuppression and tumour growth through cAMP elevation, while A_1_ and A_3_ receptors can exert anti-proliferative effects [36]. Thus, beyond their roles in neuroinflammation, adenosine A_2A_ receptors have emerged as important regulators of tumour biology. In the hypoxic tumour microenvironment, high extracellular adenosine levels chronically activate A_2A_ receptors on tumour and immune cells, promoting an immunosuppressive milieu, impairing cytotoxic T-cell function, and facilitating tumour progression and metastasis. A_2A_ receptor signalling has been linked to epithelial–mesenchymal transition, increased migration and invasion, and poor clinical outcome in several solid cancers [37,38,39,40,41]. In cervical cancer cells, adenosine acting via A_2A_/A_2B_ receptors increases PD-L1 expression and TGF-β production, further enhancing the immunosuppressive capacity of tumour cells [42]. Therefore, A_2A_ receptors not only represent a potential target for neuroprotection but also a promising node in the oncogenic signalling network, whose modulation by RSV may influence both cytoprotective and tumour-promoting processes in a context-dependent manner [37,38,39,40,41,42].

Our laboratory previously demonstrated that high-dose RSV (200 μM) exerts anti-proliferative effects in HeLa cells through A_2A_ desensitization, PKA downregulation, and A_1_ upregulation [11]. These findings raised the hypothesis that AdoR modulation contributes to RSV’s broader cytoprotective profile. The current study investigates whether low-dose, non-toxic RSV protects human cells against H_2_O_2_-induced oxidative stress and explores the contribution of adenosine receptor subtypes and Nrf2 activation to this effect in HeLa cells. In particular, we use HeLa cells as a tractable human model to dissect conserved signalling pathways, while recognizing that the direct relevance to neurodegenerative diseases requires validation in neuronal systems. Using selective antagonists and complementary assays (cell viability, ROS measurement, Nrf2 localization/expression, and Western blotting), we dissect the mechanistic interplay between RSV, AdoRs, and antioxidant pathways.

## 2. Results

### 2.1. Resveratrol Ameliorates H_2_O_2_ Toxic Effects on HeLa Cells

RSV was first tested at different concentrations to establish the dose that is beneficial but not toxic to cells, thereby ensuring its suitability for subsequent studies focused on its antioxidant properties. Hydrogen peroxide (H_2_O_2_) was applied at different concentrations to determine the adequate dose to induce oxidative damage and to evaluate whether RSV could mitigate or reverse this ROS-induced injury. Similarly, glutamate was applied to evaluate its suitability as an excitotoxicity model in HeLa cells.

**Figure 1:**
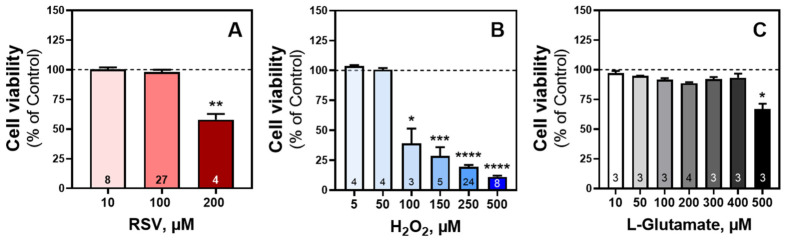
Effect of resveratrol, H_2_O_2_, and L-glutamate on HeLa cell viability. Viability was measured using the XTT assay after 24 h of treatment with the indicated concentrations of (**A**) resveratrol (RSV), (**B**) hydrogen peroxide (H_2_O_2_), or (**C**) L-glutamate. Data are presented as mean ± SEM of n independent assays (numbers within bars) performed in sextuplicate. * *p* < 0.05, ** *p* < 0.01, *** *p* < 0.001, and **** *p* < 0.0001 significantly different from control values according to Student’s *t*-test.

As shown in Figure 1A, XTT assay showed that 200 µM RSV significantly affected cell viability while 100 µM RSV was safe. This result was in line with previous studies in HeLa and other cancer cell lines, where the anti-proliferative and pro-apoptotic effects of RSV typically occur at higher concentrations (IC_50_ values around 200–250 μM for 24–48 h exposure), and lower micromolar concentrations are used for mechanistic or cytoprotective studies [11,43,44]. Therefore, the 100 µM concentration was selected for further experiments, as it provided robust cellular protection and enabled the investigation of its antioxidant activity. In contrast, H_2_O_2_ concentrations above 100 µM markedly reduced cell viability (Figure 1B), confirming its strong cytotoxic effect. Regarding the effect of glutamate on cell viability, the XTT assay showed that HeLa cells are highly resistant to glutamate-induced excitotoxicity, with a significant reduction observed only at 500 µM glutamate (Figure 1C).

**Figure 2:**
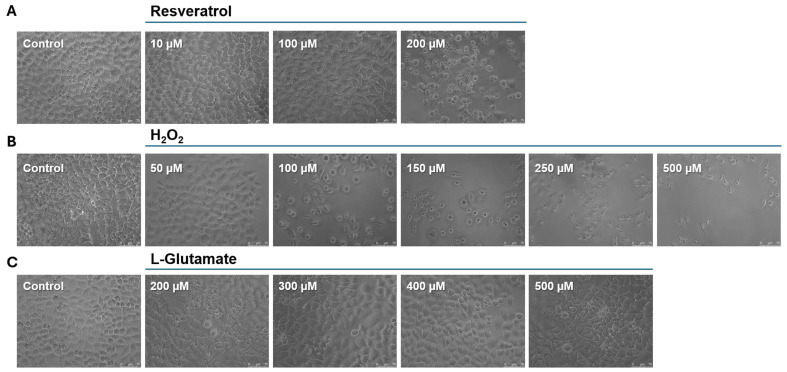
Phase-contrast imaging of HeLa cells. Phase-contrast images of HeLa cells were captured after 24 h of treatment with the indicated concentrations of (**A**) resveratrol (RSV), (**B**) hydrogen peroxide (H_2_O_2_), or (**C**) L-glutamate (L-Glu). Representative images are shown.

Phase-contrast imaging confirmed that 100 µM RSV is not harmful to cells, whereas 200 µM severely compromises cellular integrity, making this dose unsuitable for studying the antioxidant properties of RSV (Figure 2A). In contrast, hydrogen peroxide at concentrations of 100 µM or higher markedly disrupted cell morphology and integrity (Figure 2B). Glutamate treatment did not affect the cell morphology or integrity, even at high doses, and cell division was still evident at all tested concentrations (Figure 2C).

The limited susceptibility of HeLa cells to glutamate, even at high concentrations, underscores their poor suitability as a model of excitotoxicity and supported our decision to focus subsequent mechanistic analyses on H_2_O_2_-induced oxidative stress and the possible RSV-mediated cytoprotection.

**Figure 3:**
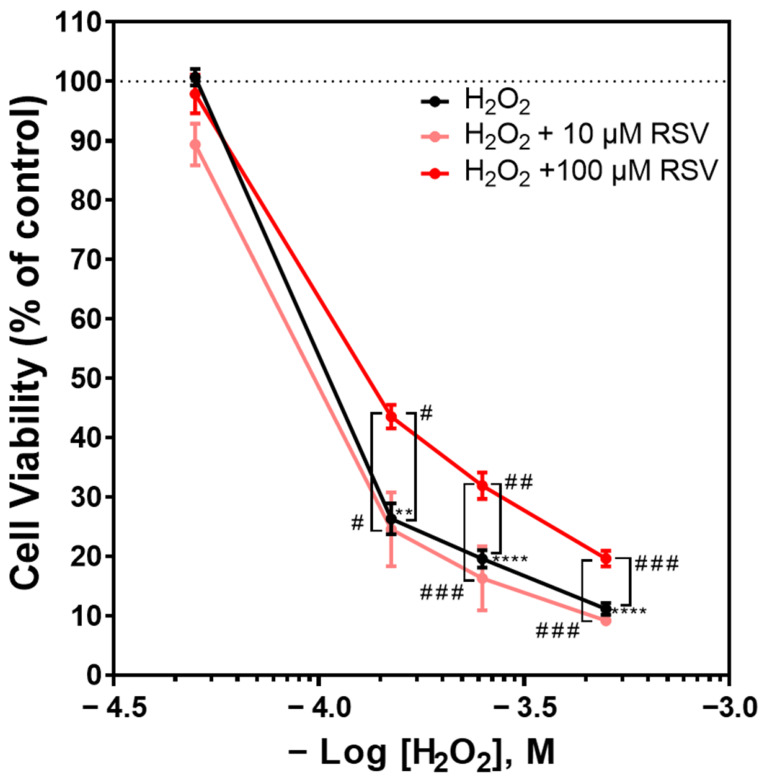
Effect of resveratrol on cell viability challenged by H_2_O_2_. Cell viability was measured using the XTT assay after treatment with 10 µM or 100 µM of resveratrol (RSV) combined with 50, 150, 250, or 500 µM H_2_O_2_. Data are presented as mean ± SEM of at least three independent assays performed in sextuplicate. ** *p* < 0.01 and **** *p* < 0.0001 significantly different from control values, according to Student’s *t*-test. # *p* < 0.05, ## *p* < 0.01, and ### *p* < 0.001 significantly different from the indicated group, according to one-way ANOVA followed by Tukey’s post hoc test.

To evaluate whether RSV could counteract the pro-oxidant effects of H_2_O_2_, HeLa cells were treated with H_2_O_2_ (50, 100, 250, and 500 µM) in the presence of either 10 or 100 µM RSV. As shown in Figure 3, H_2_O_2_ reduced HeLa cell viability in a concentration-dependent manner starting at 100 µM, while 100 µM of RSV significantly ameliorated its toxic effects.

Since 500 µM H_2_O_2_ is too high to be physiologically relevant, 250 μM H_2_O_2_ was selected for subsequent experiments as a concentration that produced a robust yet incomplete reduction in viability, thereby modelling substantial oxidative injury while preserving enough cells to detect RSV-mediated protection and to investigate underlying mechanisms. Therefore, the combination of 100 µM RSV and 250 µM H_2_O_2_ was selected to analyze the pathways involved in the antioxidant effect of RSV in HeLa cells for subsequent assays.

**Figure 4:**
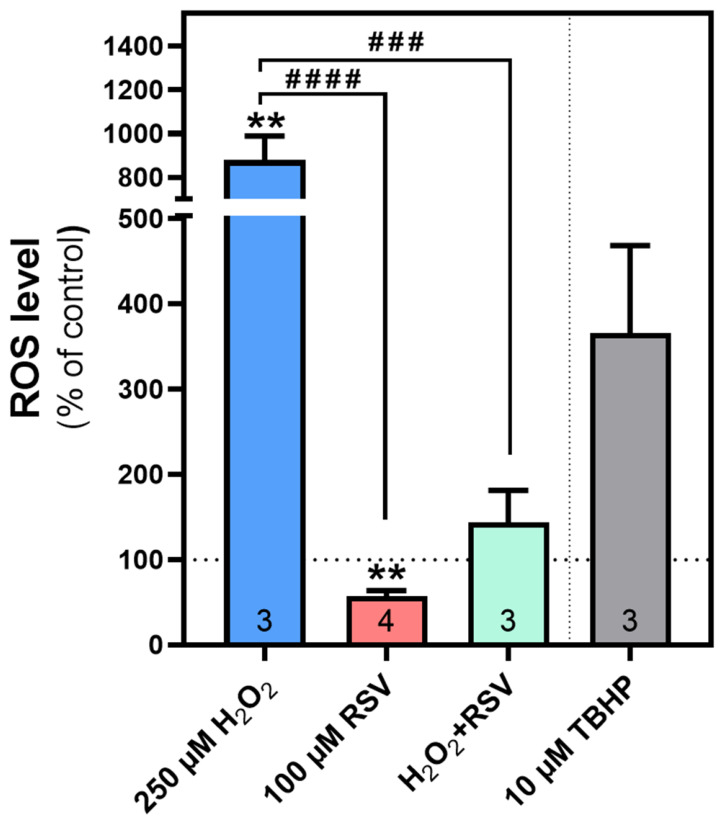
Resveratrol decreases ROS formation induced by H_2_O_2_. ROS levels were measured after 24 h of treatment with the indicated concentration of H_2_O_2_ and resveratrol (RSV), as described in Methods. Data are presented as mean ± SEM of n independent assays (numbers within bars) performed in triplicate. ** *p* < 0.01 significantly different from control values, according to Student’s *t*-test. ### *p* < 0.001, and #### *p* < 0.0001 significantly different from the indicated group, according to one-way ANOVA followed by Tukey’s post hoc test. TBHP was used as a positive control in the ROS assay.

ROS formation after H_2_O_2_ treatment was confirmed in HeLa cells (Figure 4). As expected, TBHP markedly increased DCFDA fluorescence, confirming the responsiveness of the assay to a strong pro-oxidant stimulus and validating the dynamic range used to detect changes in ROS levels induced by H_2_O_2_ and RSV. Moreover, the addition of 100 µM RSV considerably reduced H_2_O_2_-induced ROS formation, confirming the antioxidant properties of this polyphenol.

### 2.2. A_2A_ Antagonism Abolish Antioxidant Effect of RSV Against H_2_O_2_

**Figure 5:**
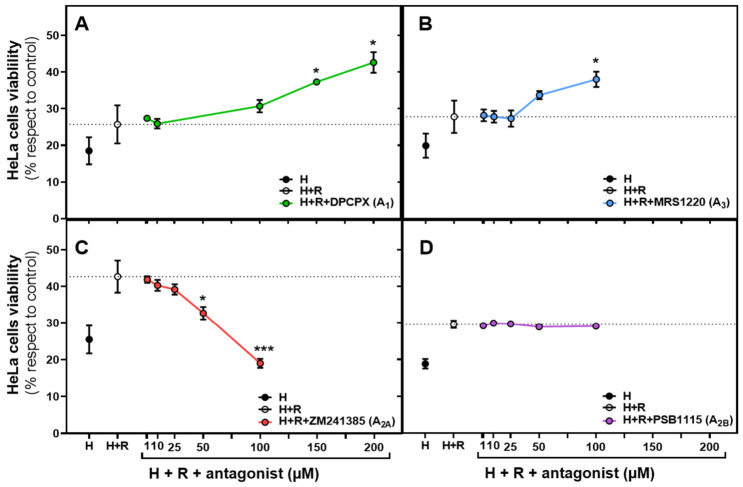
Effect of resveratrol on the decrease in cell viability induced by H_2_O_2_ after blockade of each adenosine receptor. Selective antagonists for (**A**) A_1_ (DPCPX), (**B**) A_3_ (MRS1220), (**C**) A_2A_ (ZM241385), and (**D**) A_2B_ (PSB1115) adenosine receptors were used in combination with 100 µM resveratrol and 250 µM H_2_O_2_ (H + R). The effects of 100 µM resveratrol (R) and 250 µM H_2_O_2_ (H) alone are also shown. Cell viability was assessed using the XTT assay, as indicated in Methods. Data are presented as mean ± SEM of at least five independent experiments performed in sextuplicate. * *p* < 0.05 and *** *p* < 0.001 significantly different from H + R value (dotted line), according to Student’s *t*-test.

Since RSV can bind to and modulate adenosine receptors’ signalling, its antioxidant protective effect against ROS-induced oxidative damage could be mediated, at least in part, by these receptors. To investigate this, the previously selected RSV and H_2_O_2_ combination (100 µM RSV and 250 µM H_2_O_2_) was applied to cells in the presence of antagonists for A_1_ (DPCPX), A_2A_ (ZM241385), A_2B_ (PBS1115), and A_3_ (MRS1220) receptors at different concentrations for 24 h (Figure 5). First, the adenosine A_1_ and A_3_ receptors enhanced the protective effect of RSV when blocked with DPCPX (Figure 5A) and MRS1220 (Figure 5B), respectively. Significant results were also obtained with the blockade of A_2A_ using 50 or 100 µM ZM241385 in the RSV and H_2_O_2_ combination. The antioxidant effect of RSV was abolished when A_2A_ was blocked with 100 µM ZM241385, yielding results almost identical to H_2_O_2_ treatment alone (Figure 5C), whereas A_2B_ blockade did not change the protective effect of RSV (Figure 5D). Together, these data indicate that functional A_2A_ receptor signalling is required for the full expression of RSV-associated cytoprotection under our experimental conditions, as A_2A_ blockade abolished the protective effect, whereas A_1_/A_3_ antagonism further enhanced it.

### 2.3. The Preventive Effect of RSV on H_2_O_2_-Induced Nrf2 Reduction Is Lost by Antagonizing A_2A_

Since nuclear factor erythroid 2-related factor 2 (Nrf2) is a key player in the resistance to oxidative stress, its levels were analyzed by Western blot and immunocytochemistry in HeLa cells treated with RSV or A_2A_ receptor ligands.

**Figure 6:**
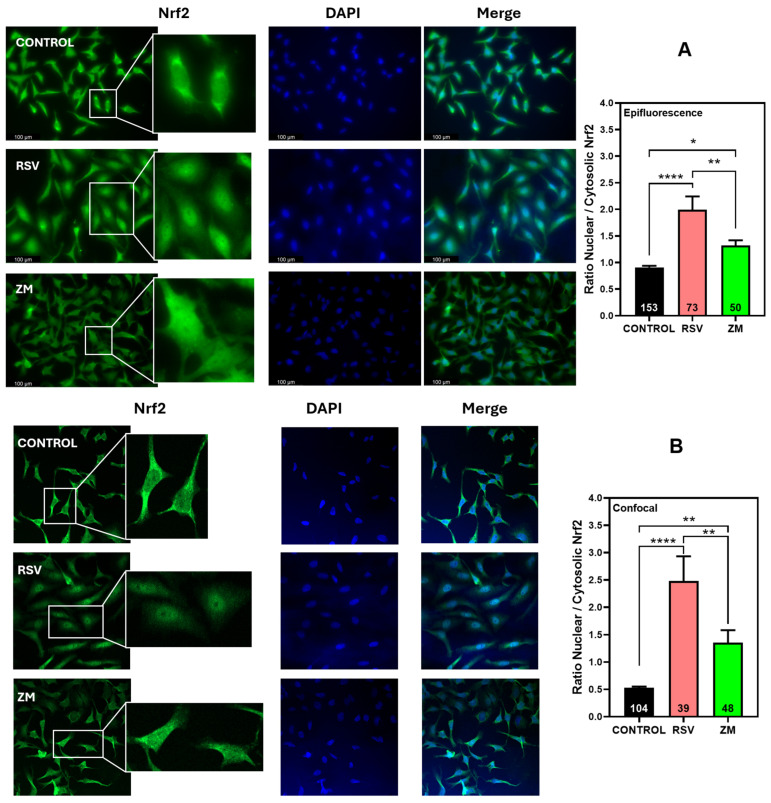
Nrf2 levels in HeLa cells. Cells were treated for 24 h with 100 µM resveratrol (RSV) or 100 µM ZM241385 (ZM). After fixation with paraformaldehyde, Nrf2 (green) and DAPI (blue) were detected, and images were acquired by epifluorescence (**A**) and confocal (**B**) microscopy, as detailed in Methods. The ratio of nuclear to cytosolic Nrf2 signal was calculated and is shown as mean ± SEM of the number of cells indicated within each bar and obtained from three different culture passages. * *p* < 0.05, ** *p* < 0.01, and **** *p* < 0.0001 significantly different from the indicated group, according to one-way ANOVA followed by Tukey’s post hoc test.

Cells treated with 100 µM RSV showed higher Nrf2 expression levels in the nuclei. Epifluorescence imaging revealed greater nuclear intensity in treated cells compared with controls, resulting in an increased nuclear-to-cytosolic ratio. A similar effect was observed after ZM241385 treatment (Figure 6A). Fluorescence co-localization analysis of Nrf2 and DAPI in confocal images confirmed a significant increase in nuclear Nrf2 levels in cells treated with 100 µM RSV or 100 µM ZM241385 to block A_2A_ receptors (Figure 6B). Moreover, the quantitative Manders’ coefficient for Nrf2-DAPI co-localization increased significantly (*p* < 0.001) from 0.417 ± 0.031 in the controls to 0.723 ± 0.021 in RSV-treated cells, confirming the enhanced nuclear presence of Nrf2 following treatment. No other antagonists were tested, since viability data clearly pointed to A_2A_ as the critical receptor subtype for resveratrol’s protective effect.

**Figure 7:**
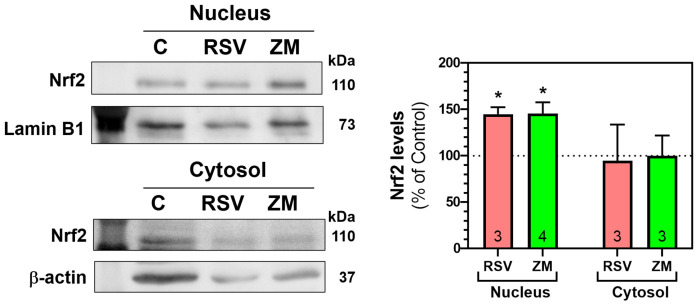
Western blot analysis of nuclear and cytosolic Nrf2 levels. Nuclear and cytosolic fractions were isolated from Hela cells after 24 h of treatment with 100 µM resveratrol (RSV) or 100 µM ZM241385 (ZM), as indicated in Methods. Data are presented as mean ± SEM of n independent assays (numbers within bars). * *p* < 0.05 significantly different from control value according to Student’s *t*-test. Lamin B1 and β-actin were used as loading controls for nuclear and cytosolic fractions, respectively.

To further confirm the increased nuclear Nrf2 levels in RSV- or ZM-treated cells, Western blot assays using an Nrf2 antibody were performed on cytosolic and nuclear fractions. As shown in Figure 7, both RSV and ZM treatments produced a similar and significant increase in Nrf2 within the nuclear fraction.

**Figure 8:**
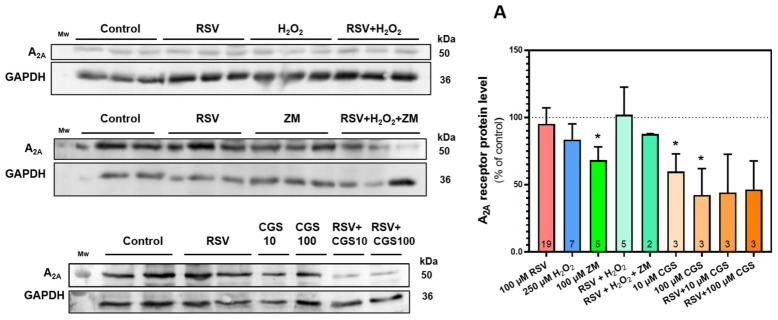

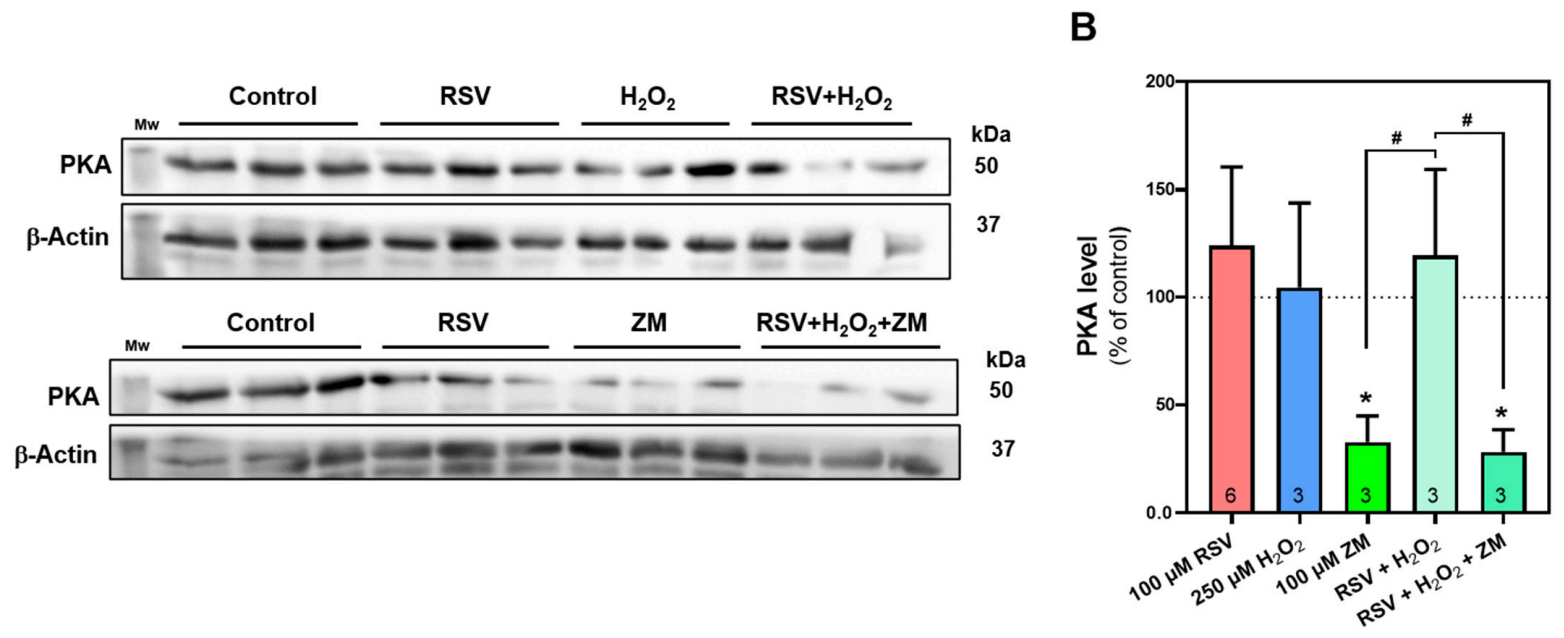
Effect on A_2A_ receptor and PKA levels. Cells were treated for 24 h with the indicated compounds before cell homogenates were isolated and subjected to SDS-PAGE for (**A**) A_2A_ receptor and (**B**) PKA immunodetection. All data are presented as mean ± SEM of n independent assays (numbers within bars). * *p* < 0.05 significantly different from control value, according to Student’s *t* test. # *p* < 0.05 significantly different from the indicated group, according to one-way ANOVA followed by Tukey’s post hoc test. ZM: ZM241385; RSV: resveratrol; and CGS: CGS21680. GAPDH and β-actin were used as loading controls for whole-cell fractions.

Neither RSV nor H_2_O_2_, nor their combination, modulated the A_2A_ receptor expression in cell homogenate. Only the A_2A_ ligands CGS21680 and ZM241385 appeared to decrease its expression (Figure 8A). Similarly, PKA expression decreased following treatment with ZM241385, an effect that was maintained in the presence of the RSV and H_2_O_2_ combination (Figure 8B).

**Figure 9:**
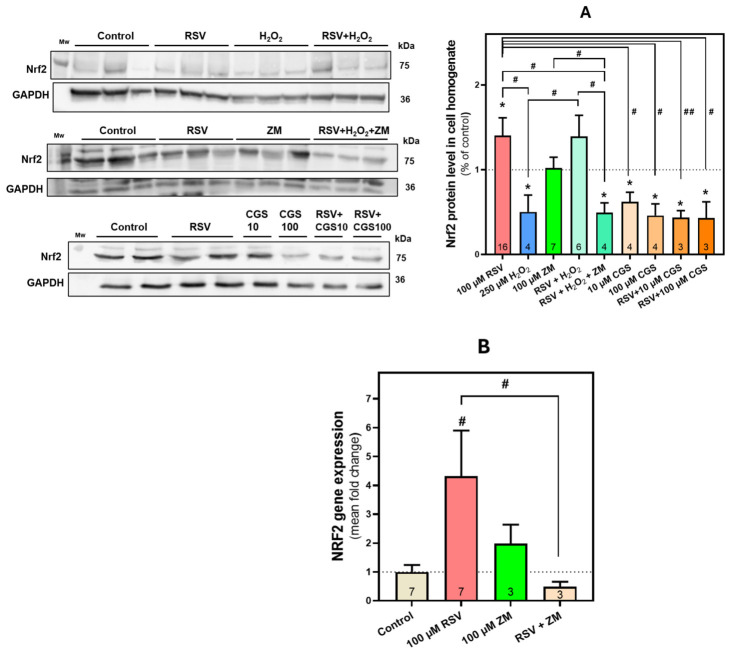
Effect on Nrf2 levels. Cells were treated for 24 h with the indicated compounds before cell homogenates and RNA were isolated and subjected to SDS-PAGE for (**A**) Nrf2 protein immunodetection and (**B**) NRF2 gene expression quantitation by real-time PCR, respectively. GAPDH was used as loading control in Western blotting assay, whereas gene expression values were normalized for β-actin relative to a calibrator, consisting of the mean expression level of the corresponding gene in control samples. All data are presented as mean ± SEM of n independent assays (numbers within bars). * *p* < 0.05 significantly different from control value, according to Student’s *t* test. # *p* < 0.05 and ## *p* < 0.01 significantly different from control or the indicated group, according to one-way ANOVA followed by Dunnett’s (**A**) or Tukey’s (**B**) post hoc test. ZM: ZM241385; RSV: resveratrol; and CGS: CGS21680.

Since Nrf2 is a transcription factor that functions as a “master switch” for cellular defence against oxidative stress and harmful substances, we assessed its protein and gene expression levels after treatments. H_2_O_2_ treatment reduced Nrf2 protein levels in cell homogenate. This reduction was prevented when cells were additionally treated with RSV, which by itself increased this protein expression. It is noteworthy that the preventive effect of RSV on H_2_O_2_-induced Nrf2 reduction is lost when the A_2A_ antagonist ZM241385 is also present. Conversely, the A_2A_ agonist CGS alone reduces Nrf2 protein levels in cell homogenate and prevents the effect of RSV when CGS and RSV are combined (Figure 9A). Nrf2 gene expression was significantly increased after 24 h treatment with 100 µM RSV (Figure 9B). The incubation of cells with ZM241385 alone did not significantly affect Nrf2 expression levels. However, ZM241385 prevented the RSV-induced upregulation of Nrf2, suggesting that the RSV effect is mediated through the A_2A_ receptor.

**Figure 10:**
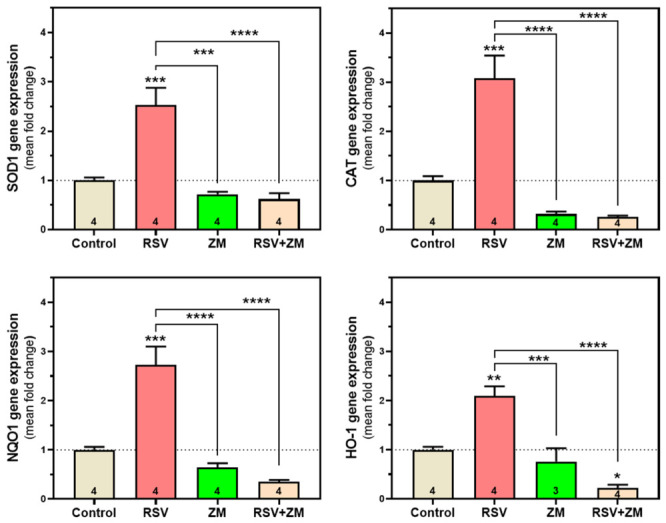
Effect on antioxidant enzymes’ gene expression. Cells were treated for 24 h with the indicated compounds before RNA were isolated and subjected to gene expression quantitation by real-time PCR for the antioxidant enzymes superoxide dismutase 1 (SOD1), catalase (CAT), NAD(P)H dehydrogenase (NQO1), and heme oxygenase-1 (HO-1). Gene expression values were normalized for β-actin relative to a calibrator, consisting of the mean expression level of the corresponding gene in control samples. All data are presented as mean ± SEM of n independent assays (numbers within bars) performed in duplicate. * *p* < 0.05, ** *p* < 0.01, *** *p* < 0.001, and **** *p* < 0.0001 significantly different from control or the indicated group, according to one-way ANOVA followed by Tukey’s post hoc test. ZM: 100 µM ZM241385; RSV: 100 µM resveratrol.

Finally, because Nrf2 regulates the expression of several antioxidant enzymes, including superoxide dismutase 1 (SOD1), catalase (CAT), NAD(P)H dehydrogenase, quinone 1 (NQO1), and heme oxygenase-1 (HO-1), their gene expression levels were quantified. Interestingly, whereas 100 µM RSV increased their expression, ZM241385 prevented the RSV-induced upregulation of all these genes (Figure 10).

## 3. Discussion

Resveratrol (RSV) at non-toxic concentrations (100 µM) exerts a clear cytoprotective effect against hydrogen peroxide (H_2_O_2_)-induced oxidative stress in HeLa cells, as demonstrated by improved cell viability, reduced ROS accumulation, and enhanced Nrf2 nuclear translocation and gene expression. The gene expression of several antioxidant enzymes such as SOD1, catalase, NQO1, and HO-1 was also increased by RSV. The H_2_O_2_ concentration range used here is consistent with many in vitro studies that use 100–500 μM H_2_O_2_ to model acute oxidative stress in mammalian cells, aiming to induce a clear injurious stimulus while avoiding immediate, near-complete cell death [45,46]. The DCFDA assay used to measure ROS levels does not allow us to distinguish between direct ROS scavenging and changes in ROS production elicited by RSV. However, on the basis of previous work, it is more likely that RSV lowers H_2_O_2_-induced oxidative stress in HeLa cells predominantly by activating endogenous antioxidant defence pathways (e.g., Nrf2-ARE signalling and induction of phase II enzymes) rather than by directly scavenging extracellular H_2_O_2_, although both mechanisms may contribute to the net reduction in ROS [17,47,48,49,50]. Neither RSV nor H_2_O_2_, nor their combination, modulated the A_2A_ receptor expression in cell homogenate. Moreover, this cytoprotective action is critically dependent on adenosine A_2A_ receptor signalling, as the selective pharmacological blockade with ZM241385 completely abolishes RSV’s antioxidant effects, whereas the antagonism of A_1_ (DPCPX) and A_3_ (MRS1220) receptors potentiates them. The observed reduction in PKA levels upon A_2A_ antagonism further implicates the cAMP/PKA signalling axis in RSV-mediated antioxidant defence.

Notably, the pharmacological activation of A_2A_ receptors with CGS21680 did not mimic the effects of RSV: CGS21680 alone reduced Nrf2 protein levels and prevented RSV-induced Nrf2 upregulation. Together with the antagonist data, this suggests that A_2A_-dependent signalling is necessary but not sufficient for RSV-associated cytoprotection in HeLa cells. In other words, the protective response appears to require the specific pattern of receptor engagement and additional pathways recruited by RSV rather than simple high-efficacy A_2A_ agonism [11,28].

Under our experimental conditions, intact A_2A_ receptor signalling is required for the full expression of RSV-associated cytoprotection and Nrf2 activation. This is consistent with the previous reports of RSV’s ability to activate Nrf2 through multiple pathways, including SIRT1-dependent deacetylation and PI3K/Akt-mediated phosphorylation [22,51,52], but also highlights that A_2A_-dependent signalling represents one contributory route rather than a simple, exclusive mechanism [53]. The requirement for A_2A_ signalling distinguishes this mechanism from direct Keap1-Nrf2 disruption and highlights adenosine receptors as novel upstream regulators of RSV’s antioxidant effects.

HeLa cells, derived from cervical carcinoma, served as a tractable human model to dissect these signalling interactions. While widely used to study conserved cellular pathways, HeLa cells lack neuronal/glial identity and brain-specific microenvironments, limiting direct extrapolation to neurodegeneration. Notably, HeLa cells exhibited marked resistance to glutamate-induced toxicity (up to 500 μM), showing no significant reduction in cell viability at glutamate concentrations typically toxic to neuronal cells [54,55], which is consistent with their low endogenous expression of ionotropic glutamate receptors and transporters [56,57,58]. This characteristic reinforces the suitability of this model for studying ROS-mediated oxidative stress rather than excitotoxicity, consistent with the study’s focus on RSV’s antioxidant action and its modulation by adenosine receptors [59].

The Nrf2 transcription factor, central to the observed cytoprotection, coordinates antioxidant gene expression through antioxidant response elements (AREs) [12]. Activation of the Nrf2 signalling pathway reduces the manifestation and severity of several human-related pathologies, including Alzheimer’s disease (AD), Parkinson’s disease, and stroke [13,14]. Some natural vegetable or plant polyphenol extracts, including RSV, have been demonstrated as Nrf2 activators [60,61]. RSV can block the repressive function of Keap1, altering the Keap1-Nrf2 interaction and enabling the stabilization and nuclear translocation of Nrf2 [17]. In addition, RSV activates multiple pathways that phosphorylate Nrf2, promoting its release from Keap1 and nuclear translocation. These include the PI3K/Akt pathway, which phosphorylates Nrf2 at specific residues to enhance its stability and transcriptional activity, and the AMPK/p38 MAPK pathway, which also facilitates Nrf2 phosphorylation and activation. Additionally, the deacetylase SIRT1, activated by RSV, indirectly modulates Nrf2 expression and function [18]. Several studies have shown that RSV upregulates HO-1, NQO1, and related Nrf2-dependent antioxidant enzymes in both neuronal models, such as PC12 cells and neural stem cells [19,47,62], and non-neuronal systems, such as endothelial and K562 cells [60,63]. In the present study, we detected the upregulation of HO-1, NQO1, SOD1, and CAT gene expression after RSV treatment. Numerous studies in other models have demonstrated that RSV can induce HO-1, NQO1, and other antioxidant enzymes via Nrf2 activation, supporting the plausibility that similar mechanisms contribute to the cytoprotective effects observed here [17,18,19,47,64,65,66]. Moreover, this effect was avoided in the presence of ZM241385, confirming the requirement of intact A_2A_ signalling for the protective effect of RSV.

The action mechanism of RSV reported here seems to be partly mediated through adenosine receptors, with a particular emphasis on the A_2A_ subtype. Experiments with selective antagonists revealed that blocking the A_2A_ receptor completely negates the protective effects of RSV, whereas antagonizing the A_1_ and A_3_ receptors appears to strengthen these effects. In contrast, inhibition of the A_2B_ receptor does not seem to influence RSV’s action. Therefore, the A_2A_ receptor pathway plays a crucial role in mediating the protective antioxidant effects of RSV in HeLa cells. Activation of this receptor influences intracellular signalling cascades that ultimately regulate the cellular response to oxidative stress, predominantly through modulation of the cAMP/protein kinase A (PKA) pathway [67,68,69]. Given that RSV is a non-selective modulator of adenosine receptors and also engages additional targets, these findings are best interpreted as evidence that A_2A_ signalling is necessary for the protective response observed here rather than as proof that RSV acts exclusively or directly through A_2A_ receptors. Interestingly, CGS21680 did not reproduce the protective profile of RSV and instead reduced Nrf2 protein levels in cell homogenates. This indicates that selective A_2A_ agonism alone is not sufficient to mimic RSV-induced cytoprotection and suggests that the outcome of A_2A_ receptor activation is highly dependent on the intensity and context of signalling rather than being uniformly associated with Nrf2 activation. Further work would be required to define the precise sequence of events downstream of RSV binding. Despite the many mechanisms involved in the many protective actions of RSV [22], the role of adenosine receptors in these actions has been scarcely studied [11,70,71,72,73]. Our research group has demonstrated the ability of RSV to act as a non-selective agonist of these receptors [28]. Moreover, we have reported, in HeLa and SH-SY5Y cell lines, that a toxic concentration of RSV (200 µM) reduces cAMP signalling by decreasing A_2A_ receptors and PKA levels while increasing A_1_ receptors and their adenylyl cyclase inhibition, contributing to the antitumoral action of RSV [11]. This opposing role of the receptors that inhibit (A_1_, A_3_) or stimulate cAMP formation (A_2A_) is again observed in this study, now in the context of the protective action of RSV used at a non-toxic concentration (100 µM), so that the blockade of A_2A_ prevents this protective effect, and the blockade of A_1_/A_3_ seems to improve it. One plausible interpretation is that, under our experimental conditions, A_1_/A_3_ signalling may partially counteract the pro-survival A_2A_-cAMP-PKA pathway engaged by RSV so that the pharmacological blockade of A_1_/A_3_ receptors shifts the balance towards A_2A_-dependent cytoprotection. This is a mechanistic hypothesis consistent with the known coupling of adenosine receptor subtypes that would require additional experiments to confirm the precise contribution of each receptor. In line with this, it has been reported that the administration of istradefylline, an A_2A_ receptor antagonist, antagonizes the beneficial effects that RSV produces on working memory and hippocampal SIRT1 expression, suggesting that RSV-mediated A_2A_ receptor activation is important for its protective action in both neural [74] and non-neural cells such as HeLa cells. Moreover, there are reports showing that A_2A_ antagonists such as SCH58261 [75] or istradefylline [76] can activate the Nrf2 pathway via mechanisms including autophagy-dependent Keap1 degradation [75]. Notably, in our experimental conditions, both RSV and, to a lesser extent, ZM241385 increased nuclear Nrf2 localisation. However, despite this shared effect on Nrf2, ZM241385 completely abolished RSV-mediated cytoprotection under oxidative stress and prevented the upregulation of canonical Nrf2 target genes such as SOD1, CAT, NQO1, and HO-1 (Figure 10). This apparent paradox suggests that A_2A_ antagonism engages a qualitatively different and functionally less efficient mode of Nrf2 activation. In line with previous work in neuronal models, A_2A_ receptor antagonists can activate the Keap1–Nrf2 pathway through autophagy-dependent Keap1 degradation [75], thereby increasing Nrf2 nuclear levels without necessarily reproducing the full cytoprotective programme observed with RSV. In our HeLa cell model, RSV appears to require intact A_2A_ receptor signalling to couple Nrf2 nuclear accumulation to a coordinated antioxidant gene response and improved cell viability, indicating that A_2A_-dependent pathways provide permissive or additional cytoprotective mechanisms beyond Nrf2 nuclear translocation alone. Thus, while both treatments increase nuclear Nrf2, the net protective efficacy depends on the specific signalling context and downstream effectors engaged under oxidative stress conditions.

The A_2A_-Nrf2 signalling axis identified here may have broader relevance to oxidative stress-related pathologies. A_2A_ receptor antagonism has been shown to activate Nrf2 through autophagy-mediated Keap1 degradation, while A_2A_ overactivation promotes pro-inflammatory signalling and glutamate release in synaptic contexts [75,77]. RSV-induced A_2A_ desensitization, as reported in prior studies from our group [28], could thus confer protection through multiple mechanisms: suppression of A_2A_-driven inflammation, relative enhancement of neuroprotective A_1_/A_3_ signalling, and liberation of Nrf2-dependent antioxidant defences.

In neurodegenerative contexts such as AD, where oxidative stress is thought to precede overt amyloid and tau pathology [14], the therapeutic potential of Nrf2 activation is well established [78,79]. However, clinical translation of RSV has been limited by age-related declines in Nrf2 responsiveness [24] and by incomplete mechanistic understanding. Importantly, the present experiments were performed exclusively in HeLa cells, a non-neuronal epithelial tumour cell line, so our findings primarily inform basic mechanisms of A_2A_-dependent regulation of resveratrol-induced Nrf2 activation and cytoprotection in this context. Our data do not allow direct conclusions about how A_2A_ antagonism would modulate resveratrol’s actions in the brain or in vivo, where cellular composition, network activity, and disease processes differ profoundly. Existing studies in neuronal and neurodegenerative models have reported context-dependent effects of A_2A_ receptor antagonists (e.g., istradefylline) on oxidative stress pathways, including both interference with resveratrol-related benefits and Nrf2 activation via autophagy-dependent Keap1 degradation [75], underscoring that A_2A_ modulation cannot be assumed to be uniformly beneficial or detrimental. Therefore, our results are best interpreted as mechanistic, hypothesis-generating evidence that A_2A_-dependent signalling can influence resveratrol-induced Nrf2 activation and cytoprotection in HeLa cells rather than as directly translatable evidence for clinical use in neurodegenerative diseases. Future studies in neuronal models (SH-SY5Y, iPSC-derived neurons) will be essential to validate this pathway’s disease relevance and explore translational applications [45,80].

From an oncological perspective, our findings need to be interpreted in the broader context of A_2A_ receptor function in cancer. A large body of evidence indicates that sustained A_2A_ activation by adenosine in the tumour microenvironment promotes immune evasion and supports tumour growth, epithelial–mesenchymal transition, and metastatic dissemination [38,39]. In cervical cancer cells, A_2A_/A_2B_ signalling has been implicated in the upregulation of PD-L1 and TGF-β, thereby increasing the immunosuppressive potential of malignant cells [42]. In contrast, the present study reveals that intact A_2A_ signalling is required for RSV-associated cytoprotection and Nrf2 activation. These short-term cytoprotective effects should not be directly extrapolated to long-term consequences for clonogenicity, motility, or invasion, which will depend on the balance between A_2A_-mediated survival pathways in tumour cells and A_2A_-driven immunosuppression in the microenvironment [38]. Future work will be necessary to determine how the chronic modulation of A_2A_ signalling by RSV influences hallmarks of cancer progression such as epithelial–mesenchymal transition, migration, and metastatic behaviour in cervical cancer models [81].

A limitation of our work is that, apart from assessing PKA protein levels as a proxy of cAMP/PKA signalling, we did not measure intracellular cAMP, Nrf2 phosphorylation, Keap1–Nrf2 interactions, or the activity of SIRT1, PI3K/Akt, or AMPK/p38 MAPK pathways, which have been implicated in RSV-induced Nrf2 activation in other models [17,48].

HeLa cells, although valuable for dissecting conserved cellular signalling pathways due to their robust transfectability and human origin, present inherent limitations as a model system. As a cervical carcinoma-derived line, HeLa cells lack neuronal identity, endogenous expression of ionotropic glutamate receptors, glutamate transporters (EAATs), and the complex neuron-glia microenvironment characteristic of brain tissue [82]. Consequently, while this model effectively identifies mechanistic interactions between RSV, adenosine A_2A_ receptors, and Nrf2 signalling, the direct extrapolation to neuroprotective effects in neurodegenerative contexts requires validation in more disease-relevant systems such as primary neurons, iPSC-derived neurons, SH-SY5Y cells, or organotypic brain slice cultures. Additionally, the immortalized nature of HeLa cells alters Nrf2-mediated redox signalling compared to primary cells [83], and A_2A_ receptor functions differ between cancer and neuronal contexts [36]. A further limitation of our study is the relatively high concentration of resveratrol used (100 μM), which exceeds the plasma levels typically attainable after oral administration in humans because of low bioavailability and extensive first-pass metabolism [84,85,86,87]. In our experimental conditions, 100 μM represented the highest non-cytotoxic concentration [11,44] that produced consistent Nrf2 activation and cytoprotection against acute H_2_O_2_-induced oxidative stress in HeLa cells, whereas lower doses elicited weaker effects, and higher doses impaired cell viability. Although clinical translation of RSV remains challenging due to pharmacokinetic limitations and biphasic dose–response relationships [26], our work identifies adenosine A_2A_ receptors as important contributors to RSV-induced cytoprotection and Nrf2 activation in HeLa cells and provides a mechanistic framework to guide future studies on adenosine–Nrf2 crosstalk in oxidative stress-related disorders. Such studies will need to employ more physiological models and improved resveratrol formulations to define dose–response relationships that are relevant for in vivo translation.

## 4. Materials and Methods

### 4.1. Cell Culture and Treatment

HeLa cells (cervical human cancer cultures) were purchased from the American Type Culture Collection (ATCC, ref. CCL-2, RRID: CVCL0030, Manassas, VA, USA) and maintained in Dulbecco’s Modified Eagle Medium (DMEM) supplemented with 10% fetal bovine serum (Biowest, ref. S181B-500, Labclinics, Madrid, Spain) and 1% antibiotic–antimycotic solution (Gibco, Carlsbad, CA, USA) in a humidified atmosphere of 95% air and 5% CO_2_ at 37 °C. Cells were exposed to different concentrations of trans-resveratrol (RSV; Sigma Aldrich, ref. R5010, Madrid, Spain), prepared from a 20 mM stock solution in 80% ethanol and subsequently diluted in culture medium to the desired concentration. The final ethanol concentration applied to the cells was ≤0.8%, which does not compromise normal cellular functionality. HeLa cells were also treated with hydrogen peroxide (H_2_O_2_; Sigma Aldrich, ref. H1009), L-glutamate (Tocris Bioscience, ref. 0218, Bristol, UK), and selective adenosine receptor (AdoR) ligands: DPCPX (A_1_ antagonist, ref. 0439), ZM241385 (A_2A_ antagonist, ref. 1036), PBS1115 (A_2B_ antagonist, ref. 2009), MRS1220 (A_3_ antagonist, ref. 1217), and CGS21680 (A_2A_ agonist, ref. 1063), all from Tocris Bioscience. Cells were treated for 24 h with 10, 100, and 200 μM RSV; 50–500 μM H_2_O_2_; 10–500 μM L-glutamate; and 1–100 μM of each AdoR ligand (DPCPX, ZM241385, PBS1115, and MRS1220).

### 4.2. Cell Viability

HeLa cells were plated in 96-well plates (3.5 × 10^4^ cells/well) and grown overnight before starting treatment. Cell numbers were determined using a TC20^TM^ Automated Cell Counter (BioRad, Madrid, Spain). Following treatment, phase-contrast images were acquired from at least 3 wells per condition, and cell viability was assessed using the XTT assay according to the manufacturer’s protocol (Roche, Madrid, Spain). Reagents were incubated for 60 min at 37 °C, and absorbances at 475 and 690 nm from six wells per condition (technical replicate) were measured using a Synergy HT plate reader (Biotek Instruments Inc., Winooski, VT, USA). Results were expressed as percentages of the control non-treated condition.

### 4.3. Phase-Contrast Imaging

Phase-contrast images of HeLa cells were captured after 24 h of treatment under all tested conditions to assess cell morphology and overall appearance. Images were taken immediately before adding the XTT reagent to the 96-well plates. At least three wells per condition were documented to evaluate the effects of different concentrations of glutamate, hydrogen peroxide, RSV, and their combinations on HeLa cells.

### 4.4. Immunocytochemistry and Fluorescence Images

HeLa cells were fixed on sterile glass chamber slides (Thermo Fisher, ref. 177402, Madrid, Spain) under selected conditions (100 μM RSV, 250 μM H_2_O_2_, 100 μM ZM241385, and their combinations), with 4% paraformaldehyde (Electron Microscopy Sciences, ref. 15715S, Morgantown, PA, USA) in PBS pH 7.2 (Thermo Fisher, ref. 20012-019) for 15 min at room temperature. After three 10 min PBS washes, cells were permeabilized for 10 min with 0.25% Triton X-100 (Sigma Aldrich, ref. T9284), followed by blocking with 10% goat serum (Vector Laboratories, ref. NC9270494) for 2 h. Slides (n ≥ 3 per antibody) were incubated overnight at 4 °C with primary anti-Nrf2 antibody (Thermo Fisher, ref. PA5-27882). After washing, Alexa Fluor 488 goat anti-rabbit IgG (Thermo Fisher, ref. A11034) was applied for 1 h at room temperature. Nuclei were counterstained with 1 μg/mL DAPI for 10 min, protected from light, and mounted with ProLong Gold antifade reagent (Invitrogen, Madrid, Spain). Nuclei, A_2A_ receptor, and Nrf2 expression were visualized and quantified by fluorescence microscopy using a DMI6000B microscope with LAS AF software (Leica Microsystems, Wetzlar, Germany) and an L5 filter. High-resolution images and 3D reconstructions of HeLa cells were additionally acquired with an LSM800 confocal microscope (Zeiss, Madrid, Spain).

### 4.5. Cell Homogenates Preparation

Cells were resuspended in RIPA buffer (10 mM MgCl_2_, 50 mM Tris-HCl, pH 7.4, 1% sodium deoxycholate, 1% NP-40, and 0.1% SDS) supplemented with protease inhibitors (100 μM PMSF and 100 μg/mL bacitracin). Samples were homogenized using a Kimble^®^ Dounce (Sigma-Aldrich) homogenizer (10 strokes with pestle A, 10 strokes with pestle B), and protein concentrations were determined using the Lowry method. Homogenates were stored at −80 °C until further analysis.

### 4.6. Western Blotting

Different isolated fractions were heated at 60 °C for 5 min and subjected to 10% SDS–polyacrylamide gel electrophoresis (SDS-PAGE) using the same amount of protein by well in all groups. After electrophoresis (120 V, 90 min), proteins were transferred to nitrocellulose membranes using the iBlot™ Dry Blotting System (Invitrogen, Barcelona, Spain). Membranes were blocked with 5% skimmed milk in PBS for 1 h to prevent nonspecific binding and then incubated overnight at 4 °C with primary antibodies: A_2A_ (1:1000, Thermo Fisher, PA1-042), Nrf2 (1:500, Thermo Fisher, PA5-27882), PKA (1:1000, Assay Biotech, C10453, San Jose, CA, USA), Lamin β1 (1:1000, Abcam, ab16048, Cambridge, UK), GAPDH (1:1000, Abcam, ab8245), and β-Actin (1:1000, Abcam, ab8226). GAPDH or β-actin were used as loading controls for whole-cell and cytosolic fractions, and Lamin B1 was used as a nuclear loading control. Nitrocellulose membranes were incubated at room temperature for 1 h with horseradish peroxidase-coupled secondary antibody (goat anti-rabbit-HRP; 1:40,000, BioRad, ref. 172-1019) under gentle agitation. Immunoreactivity was detected using the ECL Prime detection kit (GE Healthcare, Madrid, Spain) after 3 min of incubation in darkness, and images were captured with the G:Box imaging system and GeneSys 4.3 software (SynGene, Cambridge, UK).

### 4.7. Nuclei Isolation

Cell nuclei were isolated for SDS-PAGE and Western blot analysis. HeLa cells were grown to 90% confluence, washed with cold PBS, scraped, briefly centrifuged at 12,000× *g* for 10 s, and resuspended in PBS containing 0.1% NP-40 with protease inhibitors. The suspension was triturated to obtain a whole-cell lysate, which was kept on ice until sonication. The cytoplasmic fraction was collected by centrifugation at 12,000× *g* for 10 s after homogenization with 0.1% NP-40. The nuclear fraction was obtained after three consecutive centrifugation steps at 12,000× *g* for 10 s, yielding a white nuclear pellet. This pellet was resuspended in Laemmli buffer or PBS and sonicated (8 s, 16 V) using a UP200S Ultrasonic Processor (Hielscher Ultrasonics, Teltow, Germany) along with the lysate fraction. All fractions were boiled for 1 min and stored at −20 °C until use. Aliquots of the whole-cell lysate (9 μL), cytoplasmic fraction (9 μL), and nuclear fraction (3 μL) were either loaded directly onto SDS-PAGE gels or quantified using the Lowry method before Western blot analysis.

### 4.8. Quantitative Real-Time RT-PCR Analysis

Gene expression was quantified by quantitative real-time RT-PCR in an ABI Prism 7500 Fast SDS, using 20 ng of cDNA for each reaction and TaqMan Universal PCR Master Mix following the manufacturer’s indications (Applied Biosystems, Foster City, CA, USA). TaqMan probes used were Nrf2 (Hs00232352_m1), SOD1 (Hs00533490_m1), HMOX1 (Hs01110250_m1), NQO1 (Hs01045994_m1), GPx2 (Hs01591589_m1), CAT (Hs00156308_m1), and β-actin (Hs99999903_01). PCR cycling conditions were as follows: 95 °C for 20 s, followed by 40 cycles of 95 °C for 3 s and 60 °C for 30 s. Gene expression was analyzed using the 7500 Fast System SDS 1.3.1 software. Each gene expression level was normalized to its endogenous control and relativized according to a calibrator using the following equation: RQ = 2^−ΔΔCt^ = 2 ^− ((Ct target gene − Ct β-actin) sample − (Ct target gene − Ct β-actin) calibrator)^. All cDNA samples were run in duplicate, and the average represents a single value for each mRNA and sample.

### 4.9. Reactive Oxygen Species Measurement

Reactive oxygen species (ROS) formation was assessed using the DCFDA Cellular ROS Assay Kit (ab113851, Abcam, Cambridge, MA, USA). This assay employs the cell-permeant reagent 2′,7′-dichlorofluorescin diacetate (DCFDA, also known as H2DCFDA, DCFH-DA, or DCFH) to quantitatively measure ROS levels in live cells. Briefly, cells were seeded in 96-well clear bottom black plates. Confluent adherent cells were incubated in the dark at 37 °C with 20 μM DCFDA solution for 45 min. After incubation, cells were treated with the corresponding experimental conditions for 24 h. Fluorescence was measured from the top of the plate at 485 nm excitation and 535 nm emission using a Synergy HT fluorescence microplate reader (Biotek Instruments Inc.). tert-Butyl hydroperoxide (TBHP) was used as a positive control for ROS generation, as it is a well-established inducer of intracellular oxidative stress and robustly increases DCFDA fluorescence in many cell types [46].

### 4.10. Statistical and Data Analysis

Statistical analysis was performed using Student’s *t*-test or one-way ANOVA, as appropriate. Mean values were considered statistically different at *p* < 0.05. All results are shown as mean ± SEM. Statistical analyses and graph generation were conducted with GraphPad Prism 8.0 (GraphPad Software, San Diego, CA, USA). Co-localization analysis of fluorescence images was performed using Pearson correlation coefficient and Manders’ overlap coefficient with the JaCoB plugin for Image-J/FIJI 1.54p. Thresholds for statistical significance were set at Pearson r ≥ 0.6 and Manders’ coefficient ≥ 0.5. Confocal images were further analyzed using LAS-AF 2.8 software (Leica) and ZEN Blue 3.9 software (Zeiss).

## 5. Conclusions

Our findings demonstrate that RSV protects HeLa cells against hydrogen peroxide-induced oxidative stress at a non-toxic concentration (100 µM), as evidenced by improved cell viability, reduced ROS accumulation, and enhanced Nrf2 nuclear translocation and gene expression. Pharmacological inhibition of adenosine receptors indicated that A_2A_ receptor signalling is required for the full expression of this cytoprotective effect under our experimental conditions, whereas the blockade of A_1_ and A_3_ receptors further enhanced protection. Collectively, these results identify adenosine A_2A_ receptors as key modulators of RSV-associated cytoprotection in a human non-neuronal cell model. Although HeLa cells are not a disease-relevant neuronal system, these data provide mechanistic insight that can inform the design of future studies in neuronal or glial models of oxidative stress and, ultimately, neurodegeneration.

## Data Availability

The original contributions presented in this study are included in the article. Further inquiries can be directed to the corresponding author.

## References

[1] Rego A.C., Oliveira C.R. (2003). Mitochondrial dysfunction and reactive oxygen species in excitotoxicity and apoptosis: Implications for the pathogenesis of neurodegenerative diseases. Neurochem. Res..

[2] Gao G., You L., Zhang J., Chang Y.Z., Yu P. (2023). Brain Iron Metabolism, Redox Balance and Neurological Diseases. Antioxidants.

[3] Halliwell B. (2024). Understanding mechanisms of antioxidant action in health and disease. Nat. Rev. Mol. Cell Biol..

[4] Liguori I., Russo G., Curcio F., Bulli G., Aran L., Della-Morte D., Gargiulo G., Testa G., Cacciatore F., Bonaduce D. (2018). Oxidative stress, aging, and diseases. Clin. Interv. Aging.

[5] Beckman J.S., Koppenol W.H. (1996). Nitric oxide, superoxide, and peroxynitrite: The good, the bad, and ugly. Am. J. Physiol..

[6] Reddy V.P. (2023). Oxidative Stress in Health and Disease. Biomedicines.

[7] Halliwell B., Gutteridge J.M. (1995). The definition and measurement of antioxidants in biological systems. Free Radic. Biol. Med..

[8] Schieber M., Chandel N.S. (2014). ROS function in redox signaling and oxidative stress. Curr. Biol..

[9] Castagne V., Gautschi M., Lefevre K., Posada A., Clarke P.G. (1999). Relationships between neuronal death and the cellular redox status. Focus on the developing nervous system. Prog. Neurobiol..

[10] Gutteridge J.M.C., Halliwell B. (2018). Mini-Review: Oxidative stress, redox stress or redox success?. Biochem. Biophys. Res. Commun..

[11] Munoz-Lopez S., Sanchez-Melgar A., Martin M., Albasanz J.L. (2022). Resveratrol enhances A(1) and hinders A(2A) adenosine receptors signaling in both HeLa and SH-SY5Y cells: Potential mechanism of its antitumoral action. Front. Endocrinol..

[12] Ma Q. (2013). Role of nrf2 in oxidative stress and toxicity. Annu. Rev. Pharmacol. Toxicol..

[13] Gan L., Johnson J.A. (2014). Oxidative damage and the Nrf2-ARE pathway in neurodegenerative diseases. Biochim. Biophys. Acta.

[14] Bai R., Guo J., Ye X.Y., Xie Y., Xie T. (2022). Oxidative stress: The core pathogenesis and mechanism of Alzheimer’s disease. Ageing Res. Rev..

[15] Davies D.A., Adlimoghaddam A., Albensi B.C. (2021). Role of Nrf2 in Synaptic Plasticity and Memory in Alzheimer’s Disease. Cells.

[16] Bravo L. (1998). Polyphenols: Chemistry, dietary sources, metabolism, and nutritional significance. Nutr. Rev..

[17] Farkhondeh T., Folgado S.L., Pourbagher-Shahri A.M., Ashrafizadeh M., Samarghandian S. (2020). The therapeutic effect of resveratrol: Focusing on the Nrf2 signaling pathway. Biomed. Pharmacother..

[18] Kim E.N., Lim J.H., Kim M.Y., Ban T.H., Jang I.A., Yoon H.E., Park C.W., Chang Y.S., Choi B.S. (2018). Resveratrol, an Nrf2 activator, ameliorates aging-related progressive renal injury. Aging.

[19] Shen Y., Zhang M., Liu X., Jin X., Liu Z., Liu S. (2025). Resveratrol-mediated NRF2/HO-1 signaling pathway to improve postoperative cognitive dysfunction in elderly rats. Neuroreport.

[20] Kim J.H., Park E.Y., Ha H.K., Jo C.M., Lee W.J., Lee S.S., Kim J.W. (2016). Resveratrol-loaded Nanoparticles Induce Antioxidant Activity Against Oxidative Stress. Asian-Australas. J. Anim. Sci..

[21] Gote S., Dubey S., Nargund S.L., Thapa S. (2025). A systematic review of natural products targeting Nrf2-Keap1-ARE pathway and their influence on neurodegenerative disorders. Inflammopharmacology.

[22] Islam F., Nafady M.H., Islam M.R., Saha S., Rashid S., Akter A., Or-Rashid M.H., Akhtar M.F., Perveen A., Md Ashraf G. (2022). Resveratrol and neuroprotection: An insight into prospective therapeutic approaches against Alzheimer’s disease from bench to bedside. Mol. Neurobiol..

[23] Aggarwal B.B., Bhardwaj A., Aggarwal R.S., Seeram N.P., Shishodia S., Takada Y. (2004). Role of resveratrol in prevention and therapy of cancer: Preclinical and clinical studies. Anticancer Res..

[24] Franco F.N., Peixoto B.E., de Araujo G.R., Chaves M.M. (2025). Silencing of the Nrf2 pathway in aging promotes a decrease in the anti-inflammatory effect of resveratrol. Arch. Gerontol. Geriatr..

[25] Shaito A., Posadino A.M., Younes N., Hasan H., Halabi S., Alhababi D., Al-Mohannadi A., Abdel-Rahman W.M., Eid A.H., Nasrallah G.K. (2020). Potential Adverse Effects of Resveratrol: A Literature Review. Int. J. Mol. Sci..

[26] Robertson I., Wai Hau T., Sami F., Sajid Ali M., Badgujar V., Murtuja S., Saquib Hasnain M., Khan A., Majeed S., Tahir Ansari M. (2022). The science of resveratrol, formulation, pharmacokinetic barriers and its chemotherapeutic potential. Int. J. Pharm..

[27] Borea P.A., Gessi S., Merighi S., Vincenzi F., Varani K. (2018). Pharmacology of Adenosine Receptors: The State of the Art. Physiol. Rev..

[28] Sanchez-Melgar A., Albasanz J.L., Guixa-Gonzalez R., Saleh N., Selent J., Martin M. (2019). The antioxidant resveratrol acts as a non-selective adenosine receptor agonist. Free Radic. Biol. Med..

[29] Cunha R.A. (2016). How does adenosine control neuronal dysfunction and neurodegeneration?. J. Neurochem..

[30] Fredholm B.B., IJzerman A.P., Jacobson K.A., Linden J., Muller C.E. (2011). International Union of Basic and Clinical Pharmacology. LXXXI. Nomenclature and classification of adenosine receptors—An update. Pharmacol. Rev..

[31] Albasanz J.L., Perez S., Barrachina M., Ferrer I., Martin M. (2008). Up-regulation of adenosine receptors in the frontal cortex in Alzheimer’s disease. Brain Pathol..

[32] Orr A.G., Hsiao E.C., Wang M.M., Ho K., Kim D.H., Wang X., Guo W., Kang J., Yu G.Q., Adame A. (2015). Astrocytic adenosine receptor A2A and Gs-coupled signaling regulate memory. Nat. Neurosci..

[33] Canas P.M., Porciuncula L.O., Cunha G.M., Silva C.G., Machado N.J., Oliveira J.M., Oliveira C.R., Cunha R.A. (2009). Adenosine A2A receptor blockade prevents synaptotoxicity and memory dysfunction caused by beta-amyloid peptides via p38 mitogen-activated protein kinase pathway. J. Neurosci..

[34] Launay A., Nebie O., Vijaya Shankara J., Lebouvier T., Buee L., Faivre E., Blum D. (2023). The role of adenosine A(2A) receptors in Alzheimer’s disease and tauopathies. Neuropharmacology.

[35] Laurent C., Burnouf S., Ferry B., Batalha V.L., Coelho J.E., Baqi Y., Malik E., Mariciniak E., Parrot S., Van der Jeugd A. (2016). A2A adenosine receptor deletion is protective in a mouse model of Tauopathy. Mol. Psychiatry.

[36] Allard D., Turcotte M., Stagg J. (2017). Targeting A2 adenosine receptors in cancer. Immunol. Cell Biol..

[37] Deb P.K., Maity P., Sarkar B., Venugopala K.N., Tekade R.K., Batra S. (2025). Insights from Clinical Trials on A(2A) Adenosine Receptor Antagonists for Cancer Treatment. ACS Pharmacol. Transl. Sci..

[38] Han Y., Dong C., Hu M., Wang X., Wang G. (2024). Unlocking the adenosine receptor mechanism of the tumour immune microenvironment. Front. Immunol..

[39] Shi L., Wu Z., Miao J., Du S., Ai S., Xu E., Feng M., Song J., Guan W. (2019). Adenosine interaction with adenosine receptor A2a promotes gastric cancer metastasis by enhancing PI3K-AKT-mTOR signaling. Mol. Biol. Cell.

[40] Sun C., Wang B., Hao S. (2022). Adenosine-A2A Receptor Pathway in Cancer Immunotherapy. Front. Immunol..

[41] Zohair B., Chraa D., Rezouki I., Benthami H., Razzouki I., Elkarroumi M., Olive D., Karkouri M., Badou A. (2023). The immune checkpoint adenosine 2A receptor is associated with aggressive clinical outcomes and reflects an immunosuppressive tumor microenvironment in human breast cancer. Front. Immunol..

[42] Garcia-Rocha R., Monroy-Garcia A., Vazquez-Cruz A.L., Marin-Aquino L.A., Weiss-Steider B., Hernandez-Montes J., Don-Lopez C.A., Molina-Castillo G., Mora-Garcia M.L. (2024). Adenosine Increases the Immunosuppressive Capacity of Cervical Cancer Cells by Increasing PD-L1 Expression and TGF-beta Production Through Its Interaction with A(2A)R/A(2B)R. Pharmaceuticals.

[43] Zhang Y., Yuan F., Li P., Gu J., Han J., Ni Z., Liu F. (2022). Resveratrol inhibits HeLa cell proliferation by regulating mitochondrial function. Ecotoxicol. Envrion. Saf..

[44] Rodriguez-Enriquez S., Pacheco-Velazquez S.C., Marin-Hernandez A., Gallardo-Perez J.C., Robledo-Cadena D.X., Hernandez-Resendiz I., Garcia-Garcia J.D., Belmont-Diaz J., Lopez-Marure R., Hernandez-Esquivel L. (2019). Resveratrol inhibits cancer cell proliferation by impairing oxidative phosphorylation and inducing oxidative stress. Toxicol. Appl. Pharmacol..

[45] Zenin V., Ivanova J., Pugovkina N., Shatrova A., Aksenov N., Tyuryaeva I., Kirpichnikova K., Kuneev I., Zhuravlev A., Osyaeva E. (2022). Resistance to H_2_O_2_-induced oxidative stress in human cells of different phenotypes. Redox Biol..

[46] Ransy C., Vaz C., Lombes A., Bouillaud F. (2020). Use of H_2_O_2_ to Cause Oxidative Stress, the Catalase Issue. Int. J. Mol. Sci..

[47] Chen C.Y., Jang J.H., Li M.H., Surh Y.J. (2005). Resveratrol upregulates heme oxygenase-1 expression via activation of NF-E2-related factor 2 in PC12 cells. Biochem. Biophys. Res. Commun..

[48] Gu T., Wang N., Wu T., Ge Q., Chen L. (2021). Antioxidative stress mechanisms behind resveratrol: A multidimensional analysis. J. Food Qual..

[49] Zhang X., Liu X., Wan F., You W., Tan X., Sheng Q., Li C., Hu Z., Liu G., Zhao H. (2022). Protective effect of resveratrol against hydrogen peroxide-induced oxidative stress in bovine skeletal muscle cells. Meat Sci..

[50] Fukui M., Choi H.J., Zhu B.T. (2010). Mechanism for the protective effect of resveratrol against oxidative stress-induced neuronal death. Free Radic. Biol. Med..

[51] Dos Santos M.G., Schimith L.E., Andre-Miral C., Muccillo-Baisch A.L., Arbo B.D., Hort M.A. (2022). Neuroprotective Effects of Resveratrol in In Vivo and In Vitro Experimental Models of Parkinson’s Disease: A Systematic Review. Neurotox. Res..

[52] Rao Y.L., Ganaraja B., Joy T., Pai M.M., Ullal S.D., Murlimanju B.V. (2020). Neuroprotective effects of resveratrol in Alzheimer’s disease. Front. Biosci. (Elite Ed.).

[53] Chang C.P., Wu K.C., Lin C.Y., Chern Y. (2021). Emerging roles of dysregulated adenosine homeostasis in brain disorders with a specific focus on neurodegenerative diseases. J. Biomed. Sci..

[54] Moldzio R., Radad K., Krewenka C., Kranner B., Duvigneau J.C., Rausch W.D. (2013). Protective effects of resveratrol on glutamate-induced damages in murine brain cultures. J. Neural. Transm..

[55] Zhang L.N., Hao L., Wang H.Y., Su H.N., Sun Y.J., Yang X.Y., Che B., Xue J., Gao Z.B. (2015). Neuroprotective effect of resveratrol against glutamate-induced excitotoxicity. Adv. Clin. Exp. Med..

[56] Moore K.B., Mitchell C.K., Lin Y.P., Lee Y.H., Shihabeddin E., O’Brien J. (2020). Localized Calcium Signaling and the Control of Coupling at Cx36 Gap Junctions. eNeuro.

[57] Bettler B., Egebjerg J., Sharma G., Pecht G., Hermans-Borgmeyer I., Moll C., Stevens C.F., Heinemann S. (1992). Cloning of a putative glutamate receptor: A low affinity kainate-binding subunit. Neuron.

[58] Evans A.J., Gurung S., Wilkinson K.A., Stephens D.J., Henley J.M. (2017). Assembly, Secretory Pathway Trafficking, and Surface Delivery of Kainate Receptors Is Regulated by Neuronal Activity. Cell Rep..

[59] Salmaso V., Menin S., Moro S., Spalluto G., Federico S. (2025). Adenosine Receptors in Neuroinflammation and Neurodegeneration. Cells.

[60] Ungvari Z., Bagi Z., Feher A., Recchia F.A., Sonntag W.E., Pearson K., de Cabo R., Csiszar A. (2010). Resveratrol confers endothelial protection via activation of the antioxidant transcription factor Nrf2. Am. J. Physiol. Heart Circ. Physiol..

[61] Tanigawa S., Fujii M., Hou D.X. (2007). Action of Nrf2 and Keap1 in ARE-mediated NQO1 expression by quercetin. Free Radic. Biol. Med..

[62] Shen C., Cheng W., Yu P., Wang L., Zhou L., Zeng L., Yang Q. (2016). Resveratrol pretreatment attenuates injury and promotes proliferation of neural stem cells following oxygen-glucose deprivation/reoxygenation by upregulating the expression of Nrf2, HO-1 and NQO1 in vitro. Mol. Med. Rep..

[63] Hsieh T.C., Lu X., Wang Z., Wu J.M. (2006). Induction of quinone reductase NQO1 by resveratrol in human K562 cells involves the antioxidant response element ARE and is accompanied by nuclear translocation of transcription factor Nrf2. Med. Chem..

[64] Park J.S., Rustamov N., Roh Y.S. (2023). The Roles of NFR2-Regulated Oxidative Stress and Mitochondrial Quality Control in Chronic Liver Diseases. Antioxidants.

[65] Cheng A.S., Cheng Y.H., Chiou C.H., Chang T.L. (2012). Resveratrol upregulates Nrf2 expression to attenuate methylglyoxal-induced insulin resistance in Hep G2 cells. J. Agric. Food Chem..

[66] Yu X., Li X., Xu Y., Li Y., Zhou Y., Zhang J., Guo L. (2024). Resveratrol ameliorates ulcerative colitis by upregulating Nrf2/HO-1 pathway activity: Integrating animal experiments and network pharmacology. Mol. Med. Rep..

[67] Company-Marin I., Gunner J., Poyner D., Simms J., Pitt A.R., Spickett C.M. (2025). The effect of oxidative stress on the adenosine A(2A) receptor activity and signalling. Biochim. Biophys. Acta Biomembr..

[68] Nixon S.A., Lee K., Bhutta Z.A., Blanchard J., Haddad S., Hoffman S.J., Tugwell P. (2018). Canada’s global health role: Supporting equity and global citizenship as a middle power. Lancet.

[69] Stone T.W., Ceruti S., Abbracchio M.P. (2009). Adenosine receptors and neurological disease: Neuroprotection and neurodegeneration. Handbook of Experimental Pharmacology.

[70] Sanchez-Melgar A., Albasanz J.L., Palomera-Avalos V., Pallas M., Martin M. (2019). Resveratrol Modulates and Reverses the Age-Related Effect on Adenosine-Mediated Signalling in SAMP8 Mice. Mol. Neurobiol..

[71] Razali N., Agarwal R., Agarwal P., Kumar S., Tripathy M., Vasudevan S., Crowston J.G., Ismail N.M. (2015). Role of adenosine receptors in resveratrol-induced intraocular pressure lowering in rats with steroid-induced ocular hypertension. Clin. Exp. Ophthalmol..

[72] Das S., Cordis G.A., Maulik N., Das D.K. (2005). Pharmacological preconditioning with resveratrol: Role of CREB-dependent Bcl-2 signaling via adenosine A3 receptor activation. Am. J. Physiol. Heart Circ. Physiol..

[73] Gupta Y.K., Chaudhary G., Srivastava A.K. (2002). Protective effect of resveratrol against pentylenetetrazole-induced seizures and its modulation by an adenosinergic system. Pharmacology.

[74] Kasselman L.J., Renna H.A., Voloshyna I., Pinkhasov A., Gomolin I.H., Teboul I., De Leon J., Carsons S.E., Reiss A.B. (2022). Cognitive changes mediated by adenosine receptor blockade in a resveratrol-treated atherosclerosis-prone lupus mouse model. J. Tradit. Complement. Med..

[75] Sun Y., Liu C., He L. (2024). Adenosine A2A Receptor Antagonist Sch58261 Improves the Cognitive Function in Alzheimer’s Disease Model Mice Through Activation of Nrf2 via an Autophagy-Dependent Pathway. Antioxid. Redox Signal..

[76] Mihajlovic K., Dragic M., Adzic Bukvic M., Martic T., Stevanovic I., Vinit S., Bleuze M., Mansart A., Adam L., Nedeljkovic N. (2026). Dual CD73/A(2A)R blockade modulates the neurotoxic astrocyte phenotype without disrupting core inflammatory signaling. Front. Pharmacol..

[77] Dai S.S., Zhou Y.G., Li W., An J.H., Li P., Yang N., Chen X.Y., Xiong R.P., Liu P., Zhao Y. (2010). Local glutamate level dictates adenosine A2A receptor regulation of neuroinflammation and traumatic brain injury. J. Neurosci..

[78] Goel F., Singh P., Rai S.N., Yadav D.K. (2025). Nrf2/Keap1 Signaling Axis in the Brain: Master Regulator of Oxidative Stress in Neurodegenerative and Psychiatric Disorders. Mol. Neurobiol..

[79] Chu C.T., Uruno A., Katsuoka F., Yamamoto M. (2024). Role of NRF2 in Pathogenesis of Alzheimer’s Disease. Antioxidants.

[80] Iaquinta M.R., De Pace R., Benkhalqui A., Pesaresi C., Patergnani S., Righes G., Pinton P., Tognon M., Martini F., Mazzoni E. (2025). Resveratrol Affects Cell Activities, Induces Apoptosis and Regulates AMPK Signaling Pathway in Pleural Mesothelioma Cells. FASEB J..

[81] Franciosi M.L.M., do Carmo T.I.T., Zanini D., Cardoso A.M. (2022). Inflammatory profile in cervical cancer: Influence of purinergic signaling and possible therapeutic targets. Inflamm. Res..

[82] Urrestizala-Arenaza N., Cerchio S., Cavaliere F., Magliaro C. (2024). Limitations of human brain organoids to study neurodegenerative diseases: A manual to survive. Front. Cell. Neurosci..

[83] Jodar L., Mercken E.M., Ariza J., Younts C., Gonzalez-Reyes J.A., Alcain F.J., Buron I., de Cabo R., Villalba J.M. (2011). Genetic deletion of Nrf2 promotes immortalization and decreases life span of murine embryonic fibroblasts. J. Gerontol. A Biol. Sci. Med. Sci..

[84] Almeida L., Vaz-da-Silva M., Falcao A., Soares E., Costa R., Loureiro A.I., Fernandes-Lopes C., Rocha J.F., Nunes T., Wright L. (2009). Pharmacokinetic and safety profile of trans-resveratrol in a rising multiple-dose study in healthy volunteers. Mol. Nutr. Food Res..

[85] Gambini J., Ingles M., Olaso G., Lopez-Grueso R., Bonet-Costa V., Gimeno-Mallench L., Mas-Bargues C., Abdelaziz K.M., Gomez-Cabrera M.C., Vina J. (2015). Properties of Resveratrol: In Vitro and In Vivo Studies About Metabolism, Bioavailability, and Biological Effects in Animal Models and Humans. Oxid. Med. Cell. Longev..

[86] Sergides C., Chirila M., Silvestro L., Pitta D., Pittas A. (2016). Bioavailability and safety study of resveratrol 500 mg tablets in healthy male and female volunteers. Exp. Ther. Med..

[87] Smoliga J.M., Blanchard O. (2014). Enhancing the delivery of resveratrol in humans: If low bioavailability is the problem, what is the solution?. Molecules.

